# Chronic effects of an anti-angiogenic thrombospondin-1 mimetic peptide, ABT-898, on female mouse reproductive outcomes

**DOI:** 10.1186/s12958-016-0192-7

**Published:** 2016-09-07

**Authors:** Andrew K. Edwards, Irina Olariu, Diane S. Nakamura, Soo Hyun Ahn, Chandrakant Tayade

**Affiliations:** Department of Biomedical and Molecular Sciences, Queen’s University, 18 Stuart Street, Kingston, ON K7L 3N6 Canada

**Keywords:** Endometriosis, Angiogenesis, Thrombospondin-1, Reproduction

## Abstract

**Background:**

Angiogenesis is an essential process in endometriosis disease progression. Earlier, we demonstrated that anti-angiogenic peptide, ABT-898 prevents neoangiogenesis of human endometriotic lesions in a xenograft mouse model. Since angiogenesis is essential for normal ovarian and uterine function, we evaluated effects of ABT-898 on normal female reproductive processes in mice.

**Methods:**

Cycling female C57BL/6N mice were dosed with ABT-898 (100 mg/kg) or 5 % dextrose control for 21 consecutive days to cover multiple estrous cycles (average estrous cycle 4 to 5 days in mice). Pregnant female mice were dosed with ABT-898 (100 mg/kg) or control on alternate days over the course of gestation, beginning at gestation day 7.5 to 17.5 (gestation length 21 days). Histological analysis along with CD31 and Vimentin immunohistochemistry were performed on ovaries and uteri obtained from treated and control mice. To understand the influence of ABT-898 on systemic angiogenic factors, a Pro Mouse Cytokine 9-plex assay was performed on plasma samples obtained from mice prior to treatment, during the second week of ABAT-898 or control treatment and on the last day of treatment.

**Results:**

ABT-898 did not affect the number of estrous cycles over the 21 day treatment compared to control. Histological analysis of ovaries found no difference in the number of primordial, primary, secondary, and antral follicles between ABT-898 treated and control groups. Similarly, no difference was observed in the microvessel density between ABT-898 treated and control uteri, ovarian follicles or corpus luteum when assessed using CD31 or vimentin immunohistochemistry. Electron microscopy revealed similar capillary structure and appearance in both ABT-898 treated and control uteri. Although peripheral blood angiogenic cytokine profiles (IL-15, IL-18, M-CSF, b-FGF, PDGF-bb, MIG, MIP-2, LIF and VEGF) changed over the course of the intervention, there was no significant difference between ABT-898 and control groups at any of the studied time points. Treatment with ABT-898 during pregnancy had no effect on litter size at birth, pup weight at birth or pup weight at weaning.

**Conclusion:**

Our findings suggest that ABT-898 may not alter angiogenesis dependent reproductive processes in female mice. However, an extensive reproductive toxicology screening is required to substantiate use of ABT-898 in future.

**Electronic supplementary material:**

The online version of this article (doi:10.1186/s12958-016-0192-7) contains supplementary material, which is available to authorized users.

## Background

Endometriosis, the growth of endometrial glands and stroma outside of the uterus, is a disease that causes dyspareunia, dysmenorrhea, and pelvic pain. Retrograde menstruation [1] the movement of menstruating endometrial tissue from the uterus through the fallopian tubes, and into the peritoneal cavity is a widely accepted explanation for the presence of endometrium in an ectopic location [2]. Other theories include the coelomic metaplasia that postulates endometriosis arises from the metaplasia of cells lining the visceral and abdominal peritoneum and stem cell theory suggesting contributions from endometrial stem/progenitor cells and bone marrow-derived stem cells in the pathogenesis of endometriosis. The formation and survival of endometriosis lesions requires the development of a complex vascular network to meet the metabolic demands of the growing tissue. This is achieved by angiogenesis, the formation of new blood vessels from pre-existing blood vessels (reviewed in [3]). Hence, anti-angiogenic therapy is a potential promising way to medically manage endometriosis disease progression.

Angiogenesis is also essential to female reproduction. In seminal work done by Klauber et al. 1997, and Zimmerman et al., 2003, it was demonstrated that inhibition of angiogenesis in non-pregnant cycling female mice stopped endometrial maturation, corpora luteal formation and gonadotropin mediated follicle development. Inhibition of angiogenesis in pregnant female mice caused impaired decidualization, placental formation, and resulted in embryo resorption [4]. Although several anti-angiogenic compounds have been effective in limiting neovascularization of endometriotic lesions in animal models (reviewed in [5, 6]), few have been assessed for their potential negative impacts on female reproduction [7].

Thrombospondin-1 (TSP-1), an endogenous inhibitor of angiogenesis, is part of the large thrombospondin family of extracellular matrix glycoproteins [8]. It primarily affects angiogenesis by binding to CD36 on endothelial cells, inhibiting proliferation [9] and inducing apoptosis [10]. It also binds and sequesters vascular endothelial growth factor (VEGF), the primary pro-angiogenic growth factor, limiting its bioavailability [11, 12]. Using an alymphoid xenograft mouse model [13], we have previously demonstrated that the anti-angiogenic peptide ABT-898, a thrombospondin-1 (TSP-1) mimetic, prevents the neovascularization of human endometriotic lesions in mice. Mice that had previously been treated with ABT-898 were able to become pregnant, had normal decidual and placental morphology, and litter sizes indicating no lasting effects of ABT-898 on female reproduction. It was essential for us to confirm that ABT-898 was functioning to reduce lesion vascularization before proceeding to pregnancy trials.

Since angiogenesis is integral in normal physiological processes, in this study we examined the chronic effect of ABT-898 on angiogenesis dependent reproductive processes in both non-pregnant cycling and pregnant female mice. We found no evidence that ABT-898 affects endometrial or ovarian structure and function in non-pregnant cycling female mice, or litter size and pup weight of pregnant female mice.

## Methods

### Experimental design

Female C57BL/6N mice (Charles River Laboratories International, Wilmington, MA, USA), 8-weeks old were given daily 100 μL intraperitoneal injections (*n* = 11) of 100 mg/kg ABT-898 (Abbott Laboratories, Abbott Park, IL, USA) in 5 % dextrose (Baxter Corporation, Toronto, ON, Canada), or 100 μL of 5 % dextrose as a control (*n* = 11) for 21 days. After the treatment period, mice from ABT-898 treated (*n* = 8) and 5 % dextrose control (*n* = 8) groups were sacrificed, and their reproductive organs were processed for histology or electron microscopy. ABT-898 treated (*n* = 3) and 5 % dextrose control (*n* = 3) mice were also placed into breeding pairs. After the detection of a copulation plug, female mice were determined to be at gestation day 0.5. Pregnant mice were then dosed with ABT-898 or 5 % dextrose on gestation day 7.5, 9.5, 11.5, 13.5, 15.5, and 17.5. Timing for treatment during pregnancy was chosen to cover the window of important landmarks such as early placental development and immune cell enrichment as well as placental circulation. At parturition litter size and pup weight were measured, and pup weight was measured again at weaning. In order to address whether administration of ABT-898 before coitus would have any impact on the pregnancy outcomes, we performed additional set of experiments. Female C57BL/6N mice (8 week old) were treated with ABT-898 (*n* = 3) or 5 % Dextrose (*n* = 3) for 2 days (1 injection/day) and breeding pairs were set after the second injection. After detection of copulation plug, mice were injected daily with ABT-898 or 5 % dextrose intraperitoneally from gestation day 0.5 to 6.5 (gestation day 0.5 denotes presence of copulation plug). Mice were sacrificed on gestation day 7.5 for visualization and quantification of implantation sites.

### Vaginal cytology

In order to monitor the estrous cycle in ABT-898 treated and 5 % dextrose control groups, vaginal smears were collected from each mouse over the 21 day treatment period. 21 days were chose to determine impact of ABT-898 on multiple estrous cycles (average estrous cycle in mice 4–5 days). In brief, the vaginal contents were aspirated with 45 μL of PBS, which were then placed in 15 μL drops on a glass slide, and left to dry at room temperature. Dried smears were fixed with 70 % ethanol, rehydrated in deionized water, and then briefly stained with hematoxylin (Gill’s Method, Fisher Chemicals, Fair Lawn, NJ, USA), rinsed in tap water, and stained with 1 % eosin Y (Electron Microscopy Sciences, Hatfield, PA, USA). Smears were dehydrated in increasing concentration of ethanol, cleared in xylenes, and coverslipped using Permount (Electron Microscopy Sciences, Hatfield, PA, USA). Stage of the estrous cycle was determined by comparing the abundance of cornified epithelial cells, nucleated epithelial cells, and leukocytes as described in [14].

### Ovarian follicle counts

Paraformaldehyde fixed, paraffin embedded ovaries were sectioned at 5 μm and mounted on glass slides. Sections were deparaffinised using xylenes rehydrated using decreasing concentrations of ethanol, and then stained with hematoxylin and eosin as already described. In every tenth section the numbers of follicles were counted using a modified Pedersen and Peter’s classification system [15]. Only follicles that had a visible nucleus in the oocyte were counted. Follicles were classified primordial if they had a single layer of squamous granulosa cells. Primary follicles contained a single layer of cuboidal granulosa cells, secondary had two or more granulosa cell layers, and antral follicles had a visible antrum.

### Immunohistochemistry of uteri and ovaries

Paraformaldehyde fixed, paraffin embedded uteri and ovaries were sectioned at 5 μm and mounted on glass slides. Following deparaffinisation and rehydration, antigen retrieval was conducted using 0.01 M sodium citrate buffer (pH 6.0) in a hot water bath at 95 °C for 15 min. Endogenous peroxidase activity was blocked using 0.3 % hydrogen peroxide in PBS for 30 min at room temperature. Sections were blocked with 1 % bovine serum albumin in PBS for 45 min at room temperature. Primary antibody incubation (rabbit anti-mouse CD31-ab28364, or rabbit anti-mouse vimentin-ab92547, Abcam Inc., Toronto, ON, Canada) occurred overnight at 4 °C at a 1/500 dilution. Isotype antibody served as a negative control. Sections were incubated in bioinylated goat anti-rabbit secondary (1/2000 dilution, DAKO, Markham, ON, Canada) at room temperature for 60 min, then 1/4000 Extravadin-Peroxidase (Sigma Life Sciences, Oakville, ON, Canada) for 30 min at room temperature. Sections were stained with DAB+ Liquid Chromagen Substrate System (DAKO, Markham, ON, Canada) for two minutes, rinsed in deionized water, counterstained with hematoxylin (Gill’s Method), rinsed in tap water and then dehydrated, cleared and coverslipped as previously described. A semi-quantitative assessment of CD31(+) microvessel densities was done using ImageJ Pro Plus software version 6.0 (NIH, Bethesda, MD). The integrated optical density expressed in arbitrary units was calculated in three fields of view per tissue type from each animal.

### Murine peripheral blood plasma cytokine analysis

The cytokines IL-15, LIF, M-CSF, b-FGF, PDGF-bb, MIG, MIP-2, IL-18 and VEGF were measured in peripheral blood plasma using a Bioplex Pro Mouse Cytokine Group II panel 9 plex assay on a Bio-Plex 200 suspension system (both from Bio-rad Laboratories, Hercules, CA, USA) as per manufacturer’s instructions. Peripheral blood plasma was collected as previously described [13] prior to treatment, during the second week of treatment with ABT-898 or 5 % dextrose control, and on the last day of treatment. 15 μL of plasma per sample was used in the assay. Samples were diluted 4-fold. 100 μL of assay buffer and 50 μL of beads were added to the assay plate. After washing twice with 100 μL of wash buffer, 50 μL of sample was added to each well. Following 1-hour incubation (in the dark with shaking at 300RPM) wells were washed 3 times with 100 μL of wash buffer prior to adding 25 μL of detection antibody. Samples were incubated for 30 min then washed 3 times. 50 μL of streptavidin-PE was added to each well and was incubated for 10 min. After 3 wash steps, beads were resuspended in 125 μL of assay buffer, and shaken at 1100RPM for 30 seconds. The plate was read using Bio Plex 200 Suspension Array System.

### Statistical analysis

All data were analyzed using Sigma Stat 3.0 software (Systat Software, San Jose, CA, USA). Ovarian follicle counts, estrous cycles, microvessel densities and pregnancy outcomes data were analyzed using a Student’s T-Test, while peripheral blood cytokine levels where compared using a repeated measures one way ANOVA.

## Results

### The effect of ABT-898 on angiogenesis dependent reproductive processes in non-pregnant female mice

**Fig. 1:**
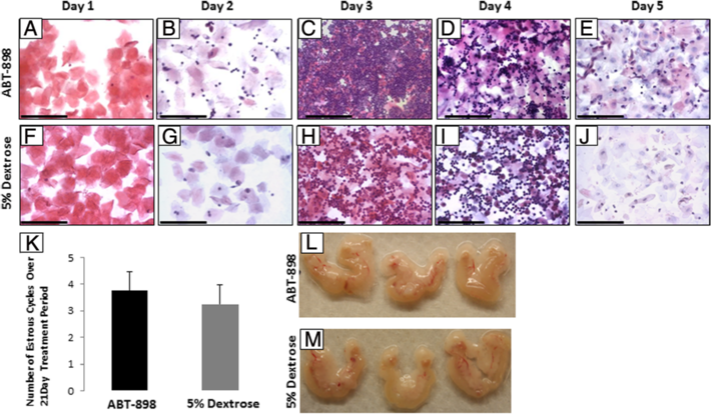
ABT-898 does not affect the estrous cycle in female mice. Representative vaginal cytology of the estrous cycle in mice dosed with ABT-898 (**a**–**e**) or 5 % dextrose (**f**–**j**). Keratinized epithelial cells (**a**, **f**) indicative of estrous were used to denote the start of a new estrous cycle. The number of estrous cycles did not significantly differ between ABT-898 and 5 % dextrose groups (3.6 vs 3.3, *p* = 0.561) over the course of the 21 day dosing period (**k**). Female reproductive tracts from mice dosed with ABT-898 (**l**) or 5 % dextrose (**m**). Gross observations indicated that female reproductive tracts from ABT-898 (**l**) treated mice did not differ from 5 % dextrose control in size (**m**), morphology or vascularity. Images (**a**–**j**) are magnified 400× and scale bars represent 75 μm

Prior to the study, to confirm that each lot of the thrombospondin-1 mimetic peptide ABT-898 had anti-angiogenic properties, we performed in vitro angiogenesis assays as previously described [13]. ABT-898 was able to completely ameliorate endothelial cell tube formation in vitro (data not shown). Non-pregnant cycling female mice were treated for 21 consecutive days with ABT-898 at a concentration (100 mg/kg) that had previously been shown to reduce neovascularization of endometriosis lesions [13], or 5 % dextrose control. Vaginal cytology was analyzed each day to assess the effect of ABT-898 on the estrous cycle. Mice showed the classical stages of the estrous cycle irrespective of treatment (representative vaginal cytology for ABT-898 Fig. 1a–e, 5 % dextrose control 1 F–J). The total number of estrous cycles did not differ between ABT-898 treated (3.75 cycles) and 5 % dextrose control groups (3.3 cycles, Fig. 1k, *p* = 0.561).

**Fig. 2:**
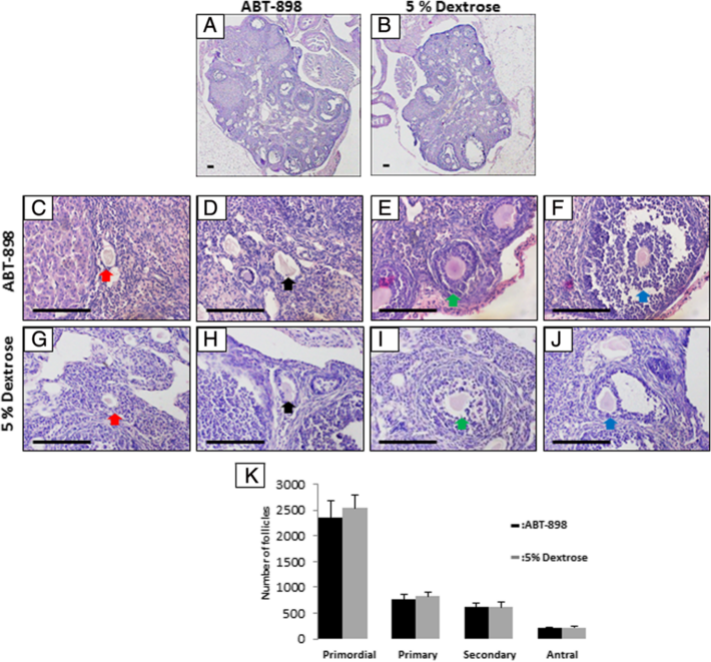
ABT-898 does not alter the number of ovarian follicles. Ovaries from mice dosed with ABT-898 (**a**) or 5 % dextrose (**b**) were sectioned and the numbers of primordial (**c**, **g**), primary (**d**, **h**), secondary (**e**, **i**) and antral (**f**, **j**) follicles were counted. The number of primordial, primary, secondary and antral follicles did not significantly differ between ABT-898 and 5 % dextrose groups (**k**). Red arrow: primordial follicle, black arrow: primary follicle, green arrow: secondary follicle, yellow arrow: antral follicle. Scale bar represents 75 μm. *P* < 0.05

After the 21 day treatment period mice were sacrificed and reproductive tracts were harvested for histological analysis. No gross differences were seen at this point in the size, structure, or vascularity of the reproductive tracts between ABT-898 (Fig. 1l), and control (Fig. 1m) groups. In an attempt to assess the effect of ABT-898 on follicular development, an angiogenesis dependent process, we performed follicle counts on ovaries from ABT-898 and control mice. There were no observable differences in the microscopic anatomy of the ovaries between ABT-898 (Fig. 2a) and control (Fig. 2b) groups. Using a modified version of Pederson’s and Peter’s classification system we counted the number of primordial, primary, secondary and antral follicles (representative follicle histology Fig. 2c–j), and found no difference between ABT-898 and control groups (Fig. 2k). These results provide initial evidence that ABT-898 does not disrupt the estrous cycle or follicular development in mice after a chronic daily treatment for 21 days.

### The effect of ABT-898 on blood vessel development and structure in reproductive organs of female mice

**Fig. 3:**
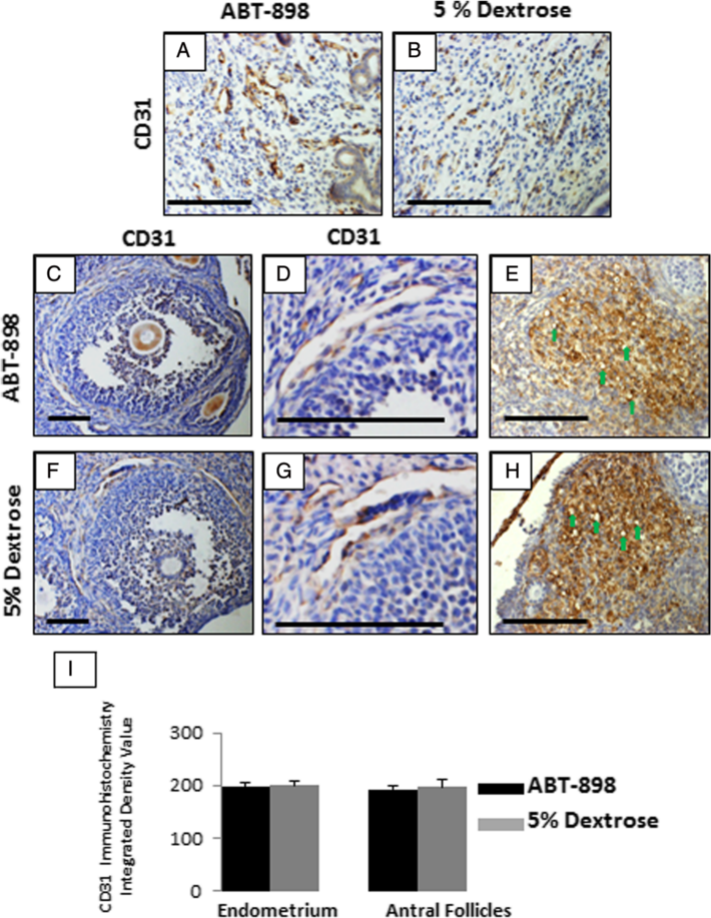
CD31 and vimentin immunohistochemistry of ABT-898 treated and 5 % dextrose control uteri and ovaries. Semi-quantitative analysis of CD31+ blood vessels in the endometrium found no significant difference (*p* = 0.45, **i**) between ABT-898 (**a**) and 5 % dextrose (**b**) groups. Immunohistochemistry for CD31 also found that blood vessel development around antral follicles was not altered by ABT-898 (**c**, **f**, **d**, **g**), and semi-quantitative analysis found no significant difference (*p* = 0.75). Vimentin immunohistochemistry showed no difference in structure or number of microvessels (green arrows) between ABT-898 (**e**) and 5 % dextrose (**h**) corpus luteum. Scale bars represent 75 μm. FOV: Field of view

We next investigated whether ABT-898 would reduce the blood vessel density, or alter endothelial cell structure in organs where physiological angiogenesis occurs, the uterus and the ovary. We performed immunohistochemistry for CD31, a pan endothelial cell marker, on ovarian and uterine sections. Semi-quantitative analysis of uterine sections found no difference in the number of CD31 (+) blood vessels between ABT-898 (Fig. 3a) and control (Fig. 3b) groups (*p* = 0.65, Fig. 3i). We also analyzed CD31 (+) blood vessels in antral follicles, and found no difference in the number or structure of blood vessels from ABT-898 (Fig. 3c, d) compared to control (Fig. 3f, g) groups (*p* = 0.803, Fig. 3i). Immunohistochemistry for vimentin, an intermediate filament expressed in cells of mesenchymal origin, was used to investigate corpora lutea. Qualitative observations found no difference in the size and structure of the corpora lutea between ABT-898 (Fig. 3e) and control (Fig. 3h) groups. Although vimentin is expressed in other cell types of the corpus luteum besides endothelial cells, blood vessels are easily identified using this stain and the numbers were similar between ABT-898 (Fig. 3e) and control (Fig. 3h) groups.

We next wanted to evaluate effect of ABT-898 on the ultra-structure of endothelial cells in reproductive organs using transmission electron microscopy. No obvious differences were seen in endothelial cells of capillary sized blood vessels of the uterus between ABT-898 (Additional file 1: Figure S1A), and control groups (Additional file 1: Figure S1B). Similar observations were made in the ovary (data not shown).

### Effect of ABT-898 on murine angiogenic cytokines in peripheral blood

**Fig. 4:**
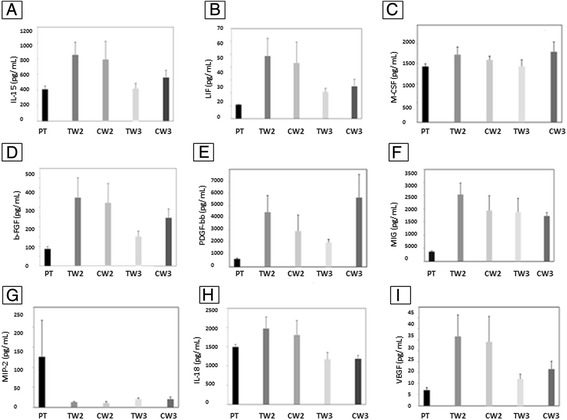
Peripheral blood plasma cytokine profiles in cycling female mice dosed with ABT-898 or 5 % dextrose control. Peripheral blood plasma levels of IL-15 (**a**), IL-18 (**b**), M-CSF (**c**), b-FGF (**d**), PDGF-bb (**e**), MIG (**f**), MIP-2 (**g**), LIF (**h**) and VEGF (**i**) did not significantly differ over the duration of the intervention or between ABT-898 and 5 % dextrose groups. PT: Pre-treatment, TW2: Treatment Week 2, CW2: Control Week 2, TW3: Treatment Week 3, CW3: Control Week 3

In an attempt to assess how ABT-898 affected angiogenesis on a systemic level, we evaluated expression of angiogenic cytokines in peripheral blood plasma over the course of the 21 day treatment using Bioplex Pro Mouse Cytokine Group II panel 9 plex angiogenesis assay. Although expression of some of the cytokines (LIF Fig. 4b; b-FGF Fig. 4d; MIP-2 Fig. 4g; VEGF Fig. 4i) differed between time points there was no statistically different expression of cytokines between ABT-898 and control groups. Taken together, these results provide preliminary evidence that ABT-898 does not impede physiological angiogenesis in the mouse ovary or uterus.

### The effect of ABT-898 on pregnancy outcomes

**Fig. 5:**
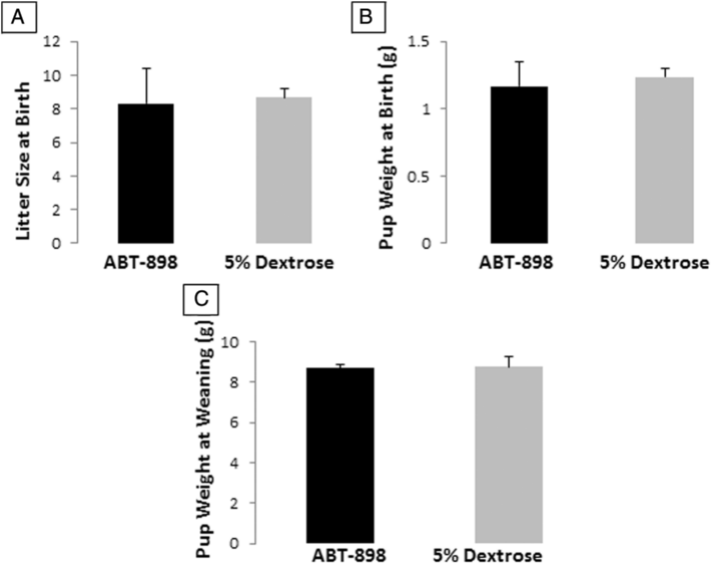
Effect of ABT-898 on mouse pregnancy outcomes. Treatment with ABT-898 on every other gestation day beginning at gestation day 7.5 did not significantly alter litter size (**a**, *p* = 0.802), pup weight at birth (**b**, *p* = 0.560), or pup weight at weaning (**c**, *p* = 0.99)

Blood vessel growth, initially through vasculogenesis and later through angiogenesis, is essential during pregnancy. We have previously shown that female mice treated with ABT-898 prior to pregnancy had normal litters and placental structures [13]. Here we wanted to establish what effect ABT-898 would have on pregnancy if it was injected chronically over the course of mouse gestation. Female mice treated with ABT-898 on alternate days over the course of gestation, beginning at gestation day 7.5, had the same litter size (Fig. 5a), pup weight at birth (Fig. 5b), and pup weight at weaning (Fig. 5c) as controls. These results provide initial evidence that ABT-898 does not affect pregnancy outcomes. Similarly, in mice treated 2 days before coitus with ABT-898 and 5 % dextrose control, allowed to get pregnant and continued treatment from gestation day 0.5 to 6.5, had comparable numbers of implantation sites between ABT-898 treated and control groups. The implantation sites in 3 ABT-898 treated mice were 7, 9 and 8. Implantation sites in 3 control mice were 9, 10 and 7. We did not observe any abnormalities in the gross examination of implantation sites between ABT-898 and control treated mice.

## Discussion

Endometriosis is a gynecological disease affecting females of child bearing age. It causes dysmenorrhea, dyspareunia, pelvic pain, and is associated with infertility [16]. Current therapeutic strategies to manage endometriosis are aimed at limiting lesion growth by preventing ovarian steroid production, and by surgical removal of lesions. These strategies often provide unsatisfactory results as recurrence rate is high after surgery (reviewed in [17]), and inhibition of ovarian steroid production does not improve fecundity [18]. New strategies are needed to manage endometriosis that limits disease progression, without affecting fertility [19]. Angiogenesis, essential in the pathogenesis of macular degeneration, diabetic retinopathy, rheumatoid arthritis, and cancer (reviewed in [20]), is also necessary for the growth and survival of endometriosis lesions. Several anti-angiogenic compounds limit the neovascularization of endometriosis in animal models; however their effects on fecundity have been poorly investigated. In this study we have demonstrated that the anti-angiogenic TSP-1 mimetic peptide, ABT-898, does not affect female mouse fertility when given chronically prior to pregnancy, or when administered during gestation. In addition, we have shown that ABT-898 does not affect the microvessel density in the ovary and uterus, where angiogenesis is essential to normal function.

Previous studies assessing the effect of ABT-898 on folliculogenesis *in vivo* using marmoset monkeys (*Callithrix jacchus*) had similar results [21]. The authors found no effect on the ovulatory cycle, and ABT-898 did not induce luteolysis [21]. Although, they saw a decrease in the number of proliferating endothelial cells in the follicles of ABT-898 treated ovaries, we did not notice any significant difference in the endothelial cells (data not shown). Our results indicate that ABT-898 did not affect microvessel density between ABT-898 treated versus control. Other reports have demonstrated that anti-angiogenic compounds are compatible with female reproduction. Becker et al., 2005 demonstrated that endostatin, an endogenous inhibitor of angiogenesis formed by the site specific cleavage of collagen XVII did not inhibit the estrous cycle, ovarian steroid production, or corpus luteum formation providing further evidence that anti-angiogenic therapies are compatible with pregnancy, at least in mouse models. While that study demonstrated that endostatin is compatible with female reproduction, we demonstrate for the first time that an anti-angiogenic agent does not reduce the blood vessel density in reproductive organs. Endostatin has a similar mechanism of action as TSP-1; both inhibit endothelial cell proliferation, induce endothelial cell apoptosis, and bind and sequester VEGF (reviewed in [22]). The similar biological properties could explain why these peptides are compatible with pregnancy, while other anti-angiogenic compounds (sFLT-1; [23], TNP-470; 4), completely inhibit female reproduction. Further research into why some anti-angiogenic compounds affect female reproduction, while others are compatible with pregnancy is needed.

We have measured pro-angiogenic cytokines including VEGF, a major controller of angiogenic processes, in systemic circulation and did not find significant differences in any of the cytokines over the period of treatment with ABT-898. TSP-1 is known to sequester VEGF and reduce its bioavailability [11], recently, Campbell et al. 2011 reported decreased levels of VEGF in the tumors from mice treated with ABT-898 treatment in a mouse model of epithelial ovarian cancer. It is possible that ABT-898 affects VEGF specifically at inflammatory sites or tumors [24] where hypoxia drives overproduction and results in imbalance in several angiogenic factors including VEGF. In normal cycling female mice, we observed similar levels of VEGF in systemic circulation between treatment and control suggesting that ABT-898 may not affect VEGF and other pro-angiogenic cytokines systemically. We have previously shown that alymphoid mice induced with endometriosis and treated for 21 days with ABT-898 were able to get pregnant at the end of the ABT-898 treatment [25]. Further treatment of alymphoid mice during pregnancy with ABT-898 did not impact litter size outcome or litter weight [25], supporting the notion that ABT-898 inhibit vascularization of endometriotic lesions without impacting reproductive outcomes in alymphoid mouse model of endometriosis [25].

We have proposed previously [13, 25] that the difference in blood vessel structure between endometriotic lesions and reproductive organs could explain why ABT-898 reduces neovascularization of endometriotic lesions, while not affecting angiogenesis in the uterus or ovary. Vasculature in pathological situations, such as endometriosis ( [26], Additional file 2: Figure S2A) and cancer [27], grow in a chaotic and disorganized manner, compared to the structured physiological neovascularization in reproductive organs (Additional file 2: Figure S2B, C). Vessels in pathological situations fail to recruit mural cells (pericytes and vascular smooth muscle cells), which provide structural support and pro-survival stimuli to endothelial cells [28]. However, we did not carry any experiments to substantiate whether endometriotic lesions indeed have issues related to pericyte recruitment and vascular stability. Although speculative, it is plausible that in the absence of mural cells, endothelial cells are more susceptible to the anti-angiogenic effects of ABT-898, compared to blood vessels in the uterus and ovary where mural cells are abundant. In this study we looked at the ultrastructure of capillary sized blood vessels in the uterus and ovary of mice treated with ABT-898. We found no difference in the ultrastructure of blood vessels between ABT-898 and control groups, giving some evidence that the structure and organization of blood vessels in reproductive organs may protect them against the anti-angiogenic effects of ABT-898. While our study provides some insights into the effects of ABT-898 on physiological angiogenesis during mouse estrous cycle and pregnancy, there are certain limitations. The sample size in the current study is small that limits scope of interpretations. Our data does not reveal why and how ABT-898 affects angiogenesis of endometriotic lesions without impacting physiological angiogenesis in the ovary and uterus. In order to delineate whether ABT-898 impacts ovarian follicular development and corpus luteum function, future work needs to be carried out in immature or hypophysectomized mice.

## Conclusion

In summary, we demonstrate for the first time that chronic administration of ABT-898 does not impact female mouse fertility. ABT-898 had no effect on the estrous cycle, ovarian follicle counts, vascularization of the uterus or ovary, or litter size and pup weight at birth. These results provide further evidence that ABT-898 may be used as a novel therapeutic agent to manage endometriosis disease progression, as it prevents neovascularization of endometriotic lesions while not affecting female fertility. However, an extensive reproductive toxicology studies are warranted in multiple species before ABT-898 can be considered for usage in human endometriosis. Our results provide first of the many steps towards evaluating ABT-898 as a future therapeutic for endometriosis.

### Supplemental methods

*Electron microscopy of the non-pregnant uterus*: Tissues were fixed by immersion in 2 % paraformaldehyde, 0.1 M sucrose and 2.5 % glutaraldehyde in PBS overnight at 4 °C. Tissues were washed five times in PBS for one minute, and then incubated in 1 % osmium tetroxide (Electron Microscopy Sciences, Hatfield, PA, USA) for 60 min at room temperature. Samples were thoroughly washed in PBS, then deionized water before dehydration in increasing concentrations of ethanol, and cleared with propylene oxide (Electron Microscopy Sciences, Hatfield, PA, USA). Sections were embedded in EMbed 812 epoxy resin (Electron Microscopy Sciences, Hatfield, PA, USA), which was polymerized at 60 °C for 36 hours. Samples were cut into ultrathin sections, mounted on formvar-coated nickel grids and stained with uranyl acetate and lead citrate. Final sections were examined using transmission electron microscopy (Hitachi 7000).

*Whole mount immunofluorescence*: Blood vessel structure of human endometriotic lesions, non-pregnant mouse uteri and ovaries was visualized using whole mount immunofluorescence (technique initially described in [29]) for CD31. Human endometriotic lesions were engrafted in alymphoid mice as previously described [13, 25]. Non-pregnant uteri and ovaries were from female C57BL/6N mice. In brief, tissues were harvested from sacrificed mice, sectioned into 1 mm^3^ pieces and fixed in 90 % methanol for two hours on ice. Sections were washed in PBS (containing 1 % bovine serum albumin (BSA), 0.1 % sodium azide and 0.3 % Triton X-100), and then incubated overnight in primary antibody (Rat anti-Mouse CD31-PE, or Mouse anti-Human CD31, both at a 1/100 dilution, BD Biosciences, Mississauga, ON, Canada) at 4 °C. The following day sections were washed twice: first in PBS (containing 1 % BSA, 0.1 % sodium azide and 0.3 % Triton X-100), followed by a subsequent wash in PBS (containing 1 % BSA and 0.1 % sodium azide). Sections were transferred to a microscope slide, coverslipped and photographed with an epifluorescence microscope using AxioVision SE64 Rel. 4.8 (Carl Zeiss Canada Ltd., Toronto, ON, Canada).

## Additional files

Additional file 1: Figure S1. (A-B)Transmission electron microscopy of non-pregnant endometrium. Ultrastructural assessment of capillary sized blood vessels in the endometrium found no obvious differences between ABT-898 (A) and 5 % dextrose groups (B). Images (A-B) are magnified 3500×. (TIF 174 kb) Additional file 2: Figure S2. Whole mount immunofluorescence for the pan endothelial cell marker CD31 in endometriotic lesions, uterus and ovary. (A) Endometriotic lesions have a robust, disorganized vascular network. (B) The mouse ovary is highly vascularized, but the blood vessels have a more uniform appearance, and an even distribution throughout the organ compared to an endometriotic lesion. (C) Anti-mesometrial view of the mouse uterus. The myometrium contains few blood vessels compared to the endometrium, which is also highly vascularized. Images are magnified 100× and scale bars represent 100 μm. AMM: Anti-mesometrial myometrium; AME: Anti-mesometrial endometrium. (TIF 510 kb)
