# Supplementary figures and images for: Chronic effects of an anti-angiogenic thrombospondin-1 mimetic peptide, ABT-898, on female mouse reproductive outcomes
Source: Reprod Biol Endocrinol. 2016 Sep 7;14(1):56. doi: 10.1186/s12958-016-0192-7 (PMC5015213; doi:10.1186/s12958-016-0192-7)

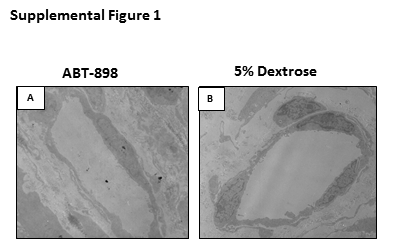

Supplement: Additional file 1: Figure S1. — (A-B)Transmission electron microscopy of non-pregnant endometrium. Ultrastructural assessment of capillary sized blood vessels in the endometrium found no obvious differences between ABT-898 (A) and 5 % dextrose groups (B). Images (A-B) are magnified 3500×. (TIF 174 kb) [file 12958_2016_192_MOESM1_ESM.tif]

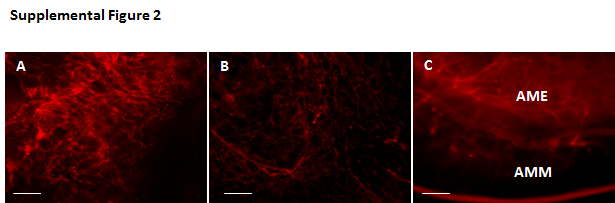

Supplement: Additional file 2: Figure S2. — Whole mount immunofluorescence for the pan endothelial cell marker CD31 in endometriotic lesions, uterus and ovary. (A) Endometriotic lesions have a robust, disorganized vascular network. (B) The mouse ovary is highly vascularized, but the blood vessels have a more uniform appearance, and an even distribution throughout the organ compared to an endometriotic lesion. (C) Anti-mesometrial view of the mouse uterus. The myometrium contains few blood vessels compared to the endometrium, which is also highly vascularized. Images are magnified 100× and scale bars represent 100 μm. AMM: Anti-mesometrial myometrium; AME: Anti-mesometrial endometrium. (TIF 510 kb) [file 12958_2016_192_MOESM2_ESM.tif]
